# γ-tocotrienol enhances the chemosensitivity of human oral cancer cells to docetaxel through the downregulation of the expression of NF-κB-regulated anti-apoptotic gene products

**DOI:** 10.3892/ijo.2012.1692

**Published:** 2012-11-08

**Authors:** KOUICHI KANI, YUKIHIRO MOMOTA, MICHITO HARADA, YOSHIKO YAMAMURA, KEIKO AOTA, TOMOKO YAMANOI, HIDEYUKI TAKANO, KATSUMI MOTEGI, MASAYUKI AZUMA

**Affiliations:** Department of Oral Medicine, Institute of Health Biosciences, The University of Tokushima Graduate Faculty of Dentistry, Tokushima 770-8504, Japan

**Keywords:** oral cancer cells, head and neck cancer, γ-tocotrienol, docetaxel, nuclear factor-κB, anti-apoptotic proteins, caspases

## Abstract

Taxanes, including docetaxel, are widely used for the treatment of squamous cell carcinoma of the head and neck. However, the gastrointestinal toxicity of docetaxel has limited its high-dose clinical use. In this study, we examined the synergistic anticancer effects of combined low-dose docetaxel and γ-tocotrienol treatment on human oral cancer (B88) cells. We treated B88 cells with docetaxel and γ-tocotrienol at concentrations of 0.5 nM and 50 μM, respectively. When cells were treated with either agent alone at a low dose, no significant cytotoxic effect was observed. However, the simultaneous treatment of cells with both agents almost completely suppressed cell growth. Whereas docetaxel stimulated the expression of nuclear factor-κB (NF-κB) p65 protein in B88 cells, γ-tocotrienol slightly inhibited the expression of constitutive nuclear p65 protein. Of note, the combined treatment with both agents inhibited docetaxel-induced nuclear p65 protein expression. Electrophoretic mobility shift assay (EMSA) revealed that the simultaneous treatment with these agents suppressed the NF-κB DNA binding activity in B88 cells. In addition, γ-tocotrienol downregulated the docetaxel-induced expression of NF-κB-regulated gene products associated with the inhibition of apoptosis. Furthermore, the activation of initiator caspases, caspases-8 and -9, and the effector caspase, caspase-3, was detected following treatment with both agents. Finally, apoptosis was also clearly observed as demonstrated by the cleavage of poly(ADP-ribose) polymerase (PARP) and nuclear fragmentation through the activation of caspase-3 by combined treatment with docetaxel and γ-tocotrienol. These findings suggest that the combination treatment with these agents may provide enhanced therapeutic response in oral cancer patients, while avoiding the toxicity associated with high-dose β-tubulin stabilization monotherapy.

## Introduction

Taxanes, including docetaxel, are widely used for the treatment of squamous cell carcinoma (SCC) of the head and neck (SCCHN), suggesting the highest single agent activity (1,2). They exert their cytotoxic effects by promoting microtubule assembly and stabilizing microtubule dynamics, thereby inhibiting cell proliferation (3). Although the clinical use of taxanes is often limited by their potentially serious side-effects, such as diarrhea, myelosuppression, mucositis and peripheral neuropathy, docetaxel has been shown to be efficacious in the treatment of patients with advanced head and neck cancer, as well as in neoadjuvant settings (4,5). However, the molecular mechanisms underlying the growth-inhibitory effect of docetaxel in SCCHN has not yet been extensively investigated.

Apoptosis (programmed cell death), plays a pivotal role in the regulation of various physiological and pathological conditions, and it is also thought to mediate chemotherapy-induced cytotoxicity (6). Apoptosis induced by chemotherapy seems to require, at least partially, the activation of the caspase cascade (7). To date, 2 major pathways leading to apoptosis have been identified: an extrinsic (receptor) pathway and an intrinsic (mitochondrial) pathway (8). The cascade led by caspase-8 (the extrinsic pathway) is involved in death-receptor-mediated apoptosis, such as the one triggered by Fas and tumor necrosis factor (TNF). Upon activation, these receptors recruit the Fas-associated death domain (FADD), which in turn binds to procaspase-8, leading to cleavage into its active form (8). The subsequent activation of the effector caspases, such as caspase-3, occurs either directly or after an amplification step involving the mitochondria (9). On the other hand, the mitochondrial (intrinsic) pathway is triggered by cytochrome *c* release from the mitochondria. Cytochrome *c* release into the cytoplasm leads to the formation of a complex with Apaf-1 that binds to procaspase-9 via its caspase recruit domain (CARD) (10). This complex, known as the apoptosome complex can, in the presence of deoxyadenosine triphosphate (dATP), activate procaspase-9, which in turn activates effector caspases including caspase-3 (11). Thus, the cascade of caspase activation plays an important role in the induction of apoptosis in cancer cells. Paradoxically, however, chemotherapeutic agents that promote apoptosis also activate the transcription factor, nuclear factor-κB (NF-κB) (12), which suppresses caspase activation by enhancing the expression of anti-apoptotic proteins, including survivin, a cellular inhibitor of apoptosis protein (cIAP)-1; cIAP-2, an X-linked inhibitor of apoptosis protein (XIAP); and B-cell lymphoma 2 (Bcl-2) (12–15). Since a human oral cancer cell line (B88) exhibited constitutively activated NF-κB activity in our previous studies (16,17), we hypothesized that the downregulation of anti-apoptotic proteins through the suppression of NF-κB activity would be a promising strategy for the treatment of patients with oral cancer.

A vitamin E constituent may be one such candidate agent derived from natural sources that can have great potential for preventing and treating oral cancer. Vitamin E is a general term representing a family of compounds that is further divided into 2 subgroups: tocopherols and tocotrienols (18). Although tocopherols and tocotrienols exist in α, β, γ and δ forms, the two differ structurally in that tocopherols contain a saturated phytyl chain, whereas tocotrienols possess an unsaturated side chain. Thus far, tocopherols have been studied extensively; however, very little is known about tocotrienols. Previous studies have clearly established that tocotrienols, but not tocopherols, display potent antiproliferative and apoptotic activity againt neoplastic mammary epithelial cells with treatment at low doses that have little or no effect on normal cell growth and function (19,20). For instance, studies have shown that γ-tocotrienol, but not tocophenol, can inhibit both constitutive and inducible NF-κB activation in various cancer cell lines (21,22). This activity correlates well with the downregulation of NF-κB-regulated gene products, such as anti-apoptotic proteins (22). Therefore, it is considered that the combined treatment with low doses of docetaxel and γ-tocotrienol may result in an enhanced therapeutic response in patients with oral cancer.

In the present study, we report that the simultaneous treatment of human oral cancer (B88) cells with low doses of docetaxel and γ-tocotrienol suppresses docetaxel-induced NF-κB activity, leading to the inhibition of the expression of anti-apoptotic proteins, which results in the activation of initiator caspases, caspase-8 and -9, as well as an effector caspase, caspase-3. We also found that these cells actually entered apoptosis, as evaluated by the cleavage of poly(ADP-ribose) polymerase (PARP) and DNA fragmentation.

## Materials and methods

### Cells and media

A metastatic human oral cancer cell line (B88) was previously established in our laboratory (23). This cell clone was cultured in DMEM (Gibco BRL, Grand Island, NY) supplemented with 10% fetal bovine serum (FBS) (Gibco) and 100 mg/ml penicillin-streptomycin (Gibco) in the presence of 5% CO_2_ in an incubator at 37°C.

### In vitro cell growth assay

Cells (5×10^3^ cells per well) were grown on 96-well plates (Falcon Labware, Lincoln Park, NJ) in DMEM supplemented with 10% serum in the presence or absence of docetaxel (0.5, 1, and 2 nM) (obtained from Sigma, St. Louis, MO) and γ-tocotrienol (50, 75, and 100 μM) (obtained from Eizai Food & Chemical Co., Tokyo, Japan, with a purity exceeding 98.7%) alone, or both for 6 days. Thereafter, 10 μl of 5 mg/ml 3-(4,5-dimethylthiazol-2-yl)-2,5-diphenyltetrazolium bromide (MTT) were added to each well and incubated for 4 h. The blue dye taken up by the cells was dissolved in dimethyl sulfodide (100 μg/ml), and the absorbance was measured with a Titertek spectrophoto meter (Flow, Irvine, UK) at 540 nm. All assays were run in triplicate.

### Nuclear and cytosolic extract preparations

The cells were seeded on 100-mm plastic petri dishes (Falcon Labware). Twenty-four hours after seeding, the cells were treated with either docetaxel, γ-tocotrienol, or both for 48 h, and nuclear extracts were then obtained according to a previously described method (24). The cells were washed twice with ice-cold PBS before being resuspended in 400 μl of ice-cold lysis buffer consisting of 10 mM N-2-hydroxyethylpiperazine-N′-2-ethane sulfonic acid (HEPES) (pH 7.9), 10 mM KCl, 0.1 mM ethylenediaminetetra-acetate (EDTA), 0.1 mM ethyleneglycolbis(b-aminoethyl ether)-N,N′-tetraacetic acid (EGTA), 0.5 mM dithiothreitol (DTT), 0.5 mg/ml benzamidine and 2 mg/ml aprotinin for 15 min. Nonidet P-40 was added to a final concentration of 0.3%, and the lysates were vortexed before being pelleted in a microfuge. The supernatants of this centrifugation were designated cytosolic extracts. Each nuclear pellet was resuspended in 50 μl of extraction buffer consisting of 10 mM HEPES (pH 7.9), 400 mM NaCl, 10 mM KCl, 0.1 mM EDTA, 0.1 mM EGTA, 1 mM DTT, 0.5 mM phenylmethylsulfonyl fluoride and 2 mg/ml aprotinin and then placed on ice for 30 min. The nuclear extracts were pelleted, and the supernatants were designated nuclear extracts. The protein concentrations contained in samples were determined by a Bio-Rad (Hercules, CA) protein assay kit.

### Labeling of oligonucleotides and electrophoretic mobility shift assay (EMSA)

The probe consisted of NF-κB-specific double-stranded oligonucleotides with the sequence 5′-AGTTGAGGGGACTTTCCCAGGC-3′ containing the κB site from the κ-light-chain enhancer in B cells. Oligonucleotides were biotin end-labeled using the biotin 3′ end labeling kit (Pierce Chemical, Rockford, IL). To extract the labeled probe, 50 μl of chloroform:isoamylalcohol (24:1) were added to each tube, followed by centrifugation briefly at 13,000 × g. The top aqueous phase containing the labeled probe was removed and saved for binding reactions. Nuclear extract proteins (5 μg) were used for EMSA with a LightShift Chemiluminescent EMSA kit (Pierce Chemical Co.) according to the manufacturer’s instructions. In brief, nuclear extract proteins were preincubated with the binding buffer for 5 min and then incubated with double-stranded, biotin-labeled consensus oligonucleotides for 15 min at room temperature. The specificity of the complex was analyzed by incubation with an excess of unlabeled competitor oligonucleotides (100-fold molar excess of labeled probe). Samples were run on 6% polyacrylamide gels. Subsequently, the DNA was transferred onto a positive nylon membrane, UV cross-linked, probed with streptavidin-HP conjugate, and incubated with the substrate of the enhanced chemiluminescence kit. The membrane was then exposed to X-ray film (Amersham Hyperfilm™ ECL, GE Healthcare Ltd., Chalfont St. Giles, UK).

### Western blot analysis of inhibitor of κB (IκB)-α, survivin, cIAP-1, cIAP-2, X-linked inhibitor of apoptosis protein (XIAP), Bcl-2, caspase-8, caspase-9, caspase-3, Apaf-1, cytochrome c, PARP and β-actin

Cytosolic extracts (20 μg) were subjected to electrophoresis on 10% SDS-polyacrylamide gels, then transferred onto nylon membranes. The membranes were blocked with 3% bovine serum albumin and incubated with each of the following antibodies (all from Cell Signaling Technology, Beverly, MA, USA): anti-IκB-α, anti-survivin, anti-cIAP-1, anti-cIAP-2, anti-XIAP, anti-Bcl-2, anti-caspase-8, anti-caspase-9, anti-caspase-3, anti-Apaf-1, anti-cytochrome *c*, anti-PARP and anti-β-actin. In the case of the detection of cytochrome *c*, possible mitochondrial contamination from the same sample was monitored using an antibody against mitochondrial cytochrome *c* oxidase subunit IV (Molecular Probes, Eugene, OR). After intervening rinses with PBS, the antibody was detected using a chemiluminescence western blot analysis kit (Amersham, Tokyo, Japan) according to the manufacturer’s instructions.

### Assessment of apoptosis induced by docetaxel and γ-tocotrienol

Cells that sloughed off the surface of the dish were collected and fixed with 1% paraformaldehyde (pH 7.4) for 10 min at room temperature. The apoptotic nuclear morphology was examined by Hoechst 33258 staining as described previously (25). Briefly, fixed cells plated on a glass slide were stained with 5 μg/ml of Hoechst 33258 (Hoechst Japan, Tokyo, Japan) for 30 min at room temperature, washed with PBS, and mounted with 80% glycerol in PBS for fluorescence microscopy.

## Results

### Cell growth inhibition by docetaxel and γ-tocotrienol

**Figure 1.**
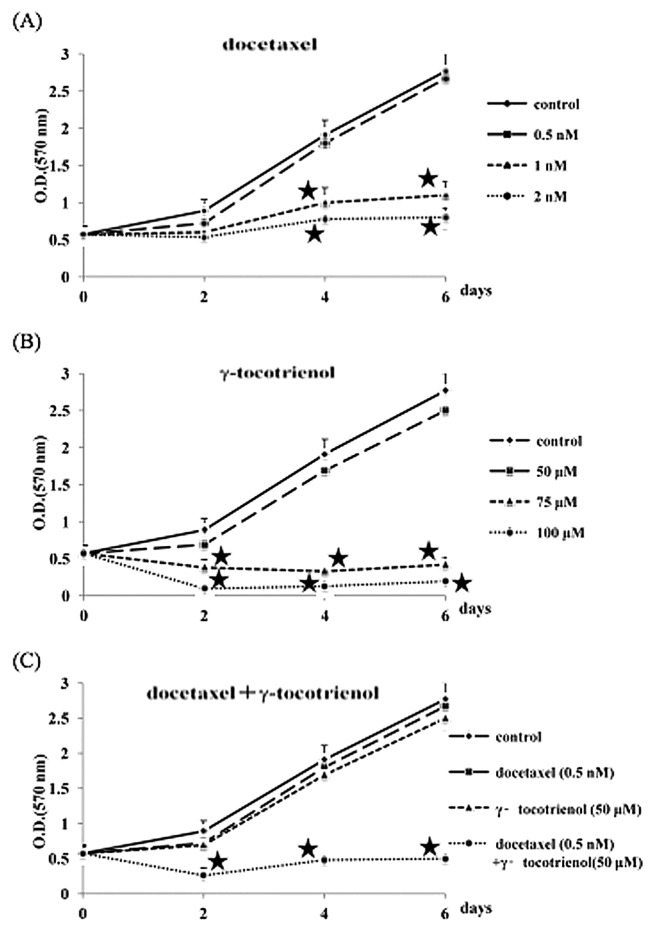
Growth suppression of human oral cancer (B88) cells by (A) docetaxel, (B) γ-tocotrienol and (C) docetaxel + γ-tocotrienol. The cells (5×10^3^ cells per well) were grown in 96-well plates in medium supplemented with docetaxel [0.5 (▪), 1 (▴) and 2 nM (•)], γ-tocotrienol [50 (▪), 75 (▴) and 100 μM (•)], or docetaxel (0.5 nM) + γ-tocotrienol (50 μM) (•) for 6 days. Viable cells were estimated by MTT assay. The absorbance was measured at 570 nm. Standard deviations were calculated from 3 independent experiments. Growth was significantly lower compared to untreated or cells treated with lower concentrations of each agent. ★, Statistically significant at P<0.05 (Mann-Whitney U test).

The growth inhibitory response of B88 cells to docetaxel and γ-tocotrienol was investigated by MTT assay for 6 days. As shown in Fig. 1 (A and B), when the number of untreated cells measured at day 6 was determined to be 100, the growth inhibitory ratios in the 0.5, 1, and 2 nM docetaxel groups were 4, 60, and 71%, respectively, and those in the 50, 75, and 100 μM γ-tocotrienol groups were 10, 85, and 93%, respectively. Therefore, we used docetaxel and γ-tocotrienol at the respective concentrations of 0.5 nM and 50 μM, neither of which significantly inhibited B88 cell growth when compared to the growth of the control cells. Whether or not γ-tocotrienol can potentiate the effect of docetaxel against B88 cells was also examined. As can be observed in Fig. 1C, the doses of docetaxel (0.5 nM) and γ-tocotrienol (50 μM) that minimally inhibited growth when used alone exhibited an enhanced suppression of cell growth when used in combination.

### γ-tocotrienol inhibition of constitutive and docetaxel-induced NF-κB activation in B88 cells

**Figure 2.**
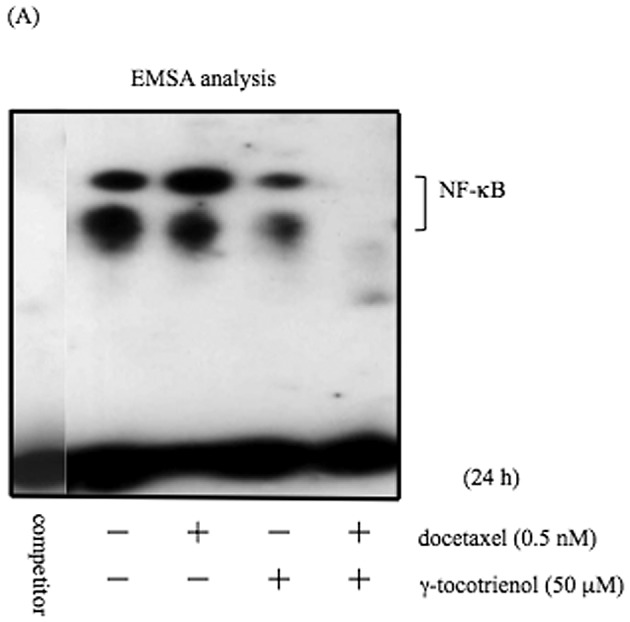

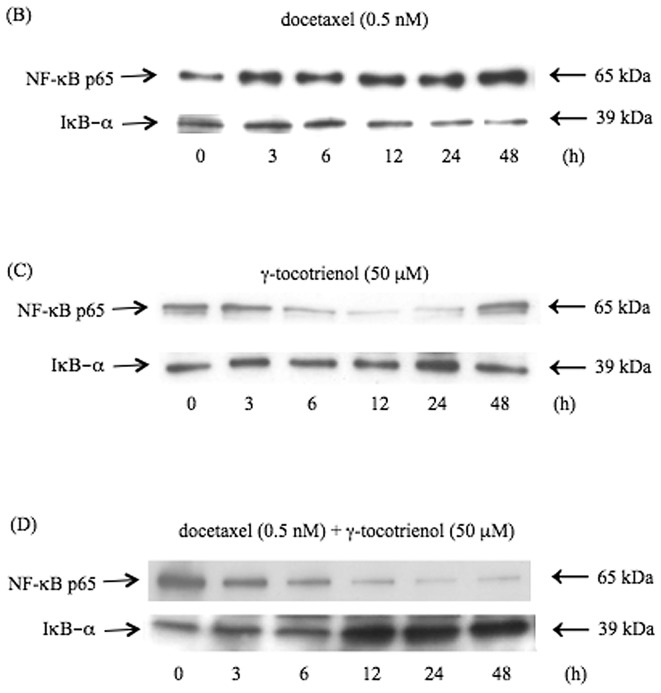
(A) Effects of docetaxel and γ-tocotrienol on the NF-κB DNA binding activity in B88 cells. Cells were treated with docetaxel (0.5 nM), γ-tocotrienol (50 μM) and docetaxel (0.5 nM) + γ-tocotrienol (50 μM) for 24 h, and nuclear extracts were prepared and analyzed by EMSA with the κB site from the κ-light-chain enhanced in B cells. The docetaxel treatment of cells stimulated the constitutive NF-κB activity, while γ-tocotrienol inhibited constitutive NF-κB activity in B88 cells. Moreover, simultaneous treatment with both agents greatly suppressed the docetaxel-induced NF-κB activity in the cells. The specificity of the complex was analyzed by incubation with an excess (100-fold) of unlabeled κB oligonucleotides (competitor). (B) Effect of docetaxel (0.5 nM) on the expression of p65 and IκB-α proteins in B88 cells. Nuclear and cytoplasmic extracts were prepared from cells following docetaxel treatment for the indicated time points and analyzed by western blot analysis. The expression of nuclear p65 was augmented by docetaxel at 12, 24 and 48 h after treatment, and cytoplasmic IκB-α expression was conversely inhibited at these time-points. (C) Effect of γ-tocotrienol (50 μM) on the expression of p65 and IκB-α proteins in B88 cells. The expression of nuclear p65 protein decreased at 12 and 24 h after treatment, and cytoplasmic IκB-α expression was conversely enhanced at these time-points. (D) When the cells were treated with both agents, a significant decrease in nuclear p65 protein expression was detected in a time-dependent manner, whereas cytoplasmic IκB-α protein expression was similarly enhanced in a time-dependent manner.

We then examined the mechanisms by which γ-tocotrienol potentiates the effects of docetaxel in B88 cells. Since various agents, including anticancer agents, have been shown to activate NF-κB through diverse pathways, we investigated the effect of γ-tocotrienol on docetaxel-induced NF-κB activation. EMSA analysis demonstrated that γ-tocotrienol suppressed the constitutively active and docetaxel-induced NF-κB activation (Fig. 2A). The specific binding of NF-κB to DNA was abrogated with an excess of unlabeled probe, indicating the NF-κB activity contained in the cells. In addition, time-course analysis of the effects of these agents on NF-κB activity in the B88 cells revealed that docetaxel alone stimulated the nuclear p65 expression at 12, 24, and 48 h after treatment. However, docetaxel inversely suppressed cytoplasmic IκB-α protein expression (Fig. 2B). As shown in Fig. 2C, when B88 cells were treated with γ-tocotrienol alone, nuclear p65 expression was significantly inhibited from 12 to 24 h after treatment. Of note, this docetaxel-induced expression of nuclear p65 was suppressed by the combined treatment with γ-tocotrienol in B88 cells, whereas cytoplasmic IκB-α protein expression was conversely enhanced (Fig. 2D). These results suggest that γ-tocotrienol is a potent modulator of both constitutive and inducible NF-κB activation in oral cancer cells.

### Effects of docetaxel and γ-tocotrienol treatment on the expression of anti-apoptotic proteins

**Figure 3.**
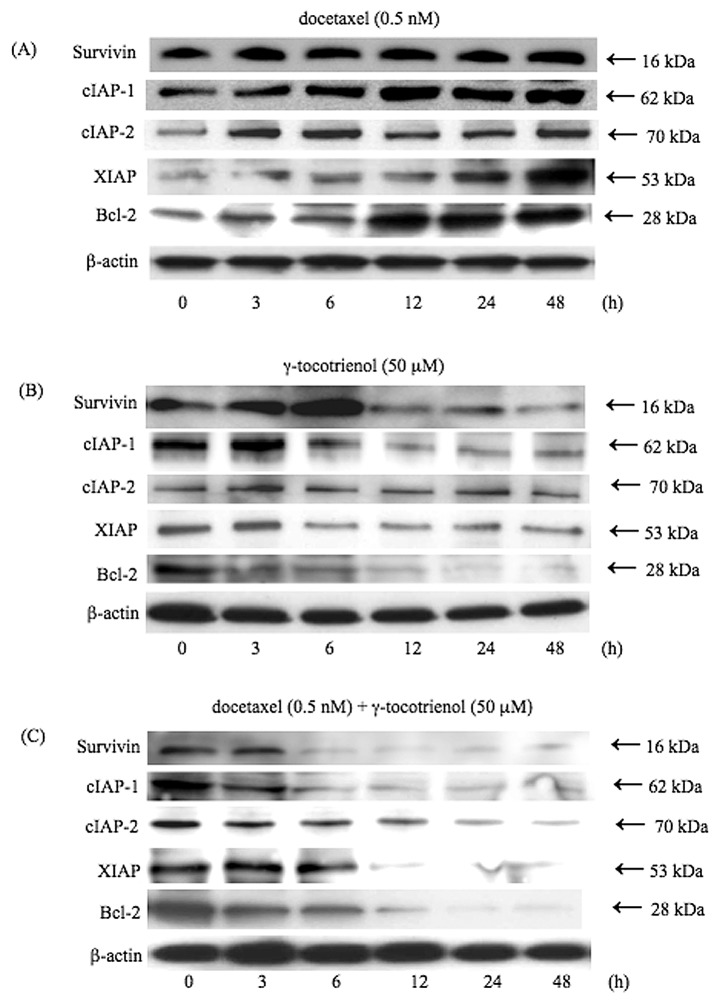
γ-tocotrienol inhibition of the anti-apoptotic gene products, survivin, cIAP-1, cIAP-2, XIAP and Bcl-2. Cells were left untreated or incubated with either (A) docetaxel (0.5 nM), (B) γ-tocotrienol (50 μM), or (C) both agents for 48 h. Whole-cell extracts were prepared and were analyzed by western blot analysis using antibodies against survivin, cIAP-1, cIAP-2, XIAP, Bcl-2 and β-actin as indicated. Although docetaxel stimulated the expression of these anti-apoptotic proteins, γ-tocotrienol slightly suppressed the expression of these proteins. On the other hand, simultaneous treatment of cells with both agents significantly inhibited the expression of anti-apoptotic proteins. As a loading control for the protein samples, the expression of β-actin is demonstrated. Results are representative of 3 independent experiments.

Since NF-κB regulates the expression of several anti-apoptotic proteins, including survivin, cIAP-1, cIAP-2, XIAP, and Bcl-2 (13–15,26–28), we examined the expression levels of these anti-apoptotic proteins by treatment with docetaxel, γ-tocotrienol, or both. As shown in Fig. 3, although docetaxel induced the expression of these anti-apoptotic proteins in a time-dependent manner (Fig. 3A), the inhibitory effects on the expression of anti-apoptotic proteins were observed in the γ-tocotrienol-treated cells (Fig. 3B). However, the simultaneous treatment of cells with docetaxel and γ-tocotrienol led to the downregulation of the expression of these anti-apoptotic proteins in a time-dependent manner, in agreement with the degree of inhibition of NF-κB activity (Fig. 3C).

### Processing of caspases-3, -8, and -9 by docetaxel and γ-tocotrienol

**Figure 4.**
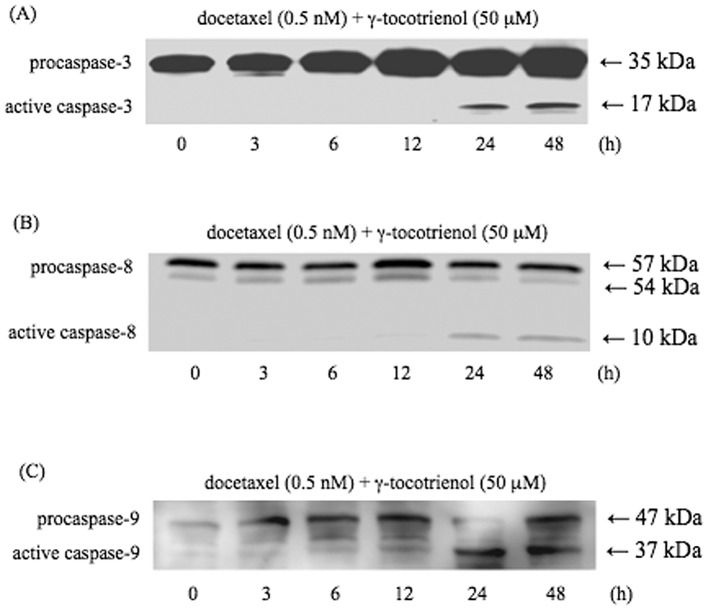
Induction of processing of initiator caspases and effector caspase by docetaxel and γ-tocotrienol. (A) Induction of caspase-3 processing by agents in B88 cells. The activation of caspase-3 was clearly observed at 24 and 48 h after treatment. Results are representative of 3 independent experiments. (B) Activation of caspase-8, as detected by cleavage of procaspase-8, was also evident after treatment with these agents. Results are representative of 3 independent experiments. (C) Induction of caspase-9 processing by agents. Docetaxel and γ-tocotrienol induced the activation of caspase-9. Results are representative of 3 independent experiments.

To understand the mechanisms involved in the enhanced cytotoxicity of the combined treatment with docetaxel and γ-tocotrienol, we analyzed the apoptotic cascades, including the processing of procaspases-3, -8 and -9, the release of cytochrome *c* from the mitochondria, and the induction of Apaf-1 by using western blot analysis. Since we have previously shown that in the apoptotic pathway, caspase-3 is a downstream caspase responsible for the cleavage of important substrates such as PARP in B88 cells (16), we first examined the processing of caspase-3 by docetaxel and γ-tocotrienol. As shown in Fig. 4A, when administered simultaneously, both agents converted procaspase-3 with a molecular weight of 35 kDa into active caspase-3 with a molecular weight of 17 kDa. To analyze the processing of procaspase-8, which stimulates the release of cytochrome *c* from the mitochondria (29), cytosolic extracts obtained from the treated cells were subjected to western blot analysis. As shown in Fig. 4B, procaspase-8 with a molecular weight of 57 kDa and 54 kDa was cleaved to active species with a molecular weight of 10 kDa. To further examine the processing of procaspase-9 mediated by cytochrome *c*, cytosolic extracts were subjected to western blot analysis. As shown in Fig. 4C, when B88 cells were treated with both agents, procaspase-9 with a molecular weight of 47 kDa was converted to an active form of caspase-9 with a molecular weight of 37 kDa.

### Induction of Apaf-1 and cytochrome c release by docetaxel and γ-tocotrienol

**Figure 5.**
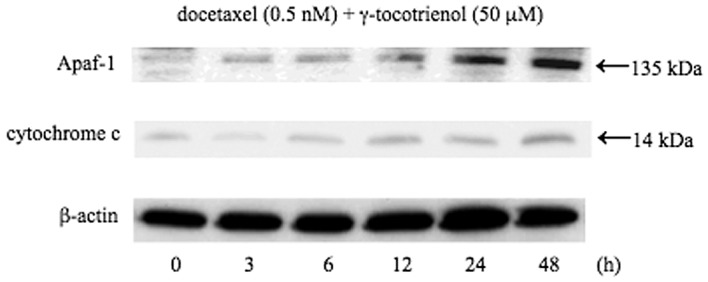
Western blot analysis for cytochrome *c* and Apaf-1 expression in cytosolic extracts in B88 cells. Cells were treated with docetaxel (0.5 nM) and γ-tocotrienol (50 μM) for the indicated periods of time, and cytosolic extracts were subjected to analysis. Both agents induced the release of cytochrome *c* into the cytoplasm. To ensure the lack of mitochondrial contamination of the extracts, the membrane was also probed with an antibody against mitochondrial cytochrome *c* oxidase subunit IV (data not shown). The treatment of cells with docetaxel and γ-tocotrienol also induced the expression of Apaf-1. As a loading control for the protein samples, the expression of β-actin is demonstrated. Results are representative of 3 independent experiments.

The initiation of mitochondria-mediated apoptosis requires the release of cytochrome *c* into the cytoplasm, where a complex (apoptosome) is formed with procaspase-9, Apaf-1 and dATP (10). The release of cytochrome *c* into the cytoplasm is thought to be the limiting factor in caspase-9 activation and the subsequent activation of caspase-3 (10). Thus, we assessed cytochrome *c* release into the cytoplasm in response to docetaxel and γ-tocotrienol in B88 cells. The results shown in Fig. 5 demonstrate that simultaneous treatment with these agents induced the release of cytochrome *c* into the cytoplasm in a time-dependent manner. Apaf-1, another component of the apoptosome complex, was also augmented in the agent-treated B88 cells.

### Cleavage of PARP by docetaxel and γ-tocotrienol

**Figure 6.**
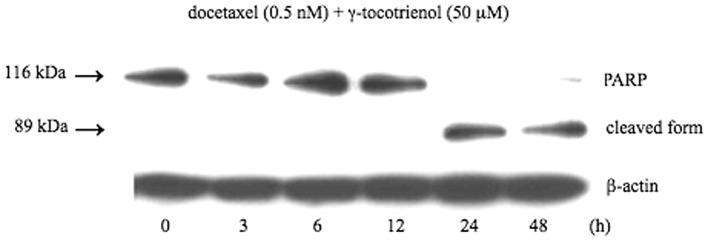
Western blot analysis of PARP cleavage in B88 cells treated with the combination of docetaxel (0.5 nM) and γ-tocotrienol (50 μM) for 48 h. Whole-cell fractions extracted from the cells were subjected to analysis. The molecular weights of uncleaved and cleaved PARP were 116 and 89 kDa, respectively. Results are representative of 3 independent experiments.

PARP is a 116-kDa nuclear enzyme that detects and binds DNA strand breaks produced by various apoptotic stimuli. It is known as a substrate for caspase-3. Caspase-3 mediated PARP cleavage into a molecular weight of 89 kDa is considered a hallmark of apoptosis (30). Therefore, we investigated the cleavage of PARP by both agents. As shown in Fig. 6, increased cleavage of PARP was observed at 24 and 48 h following treatment with both agents.

### Induction of apoptosis in B88 cells treated with docetaxel and γ-tocotrienol

**Figure 7.**
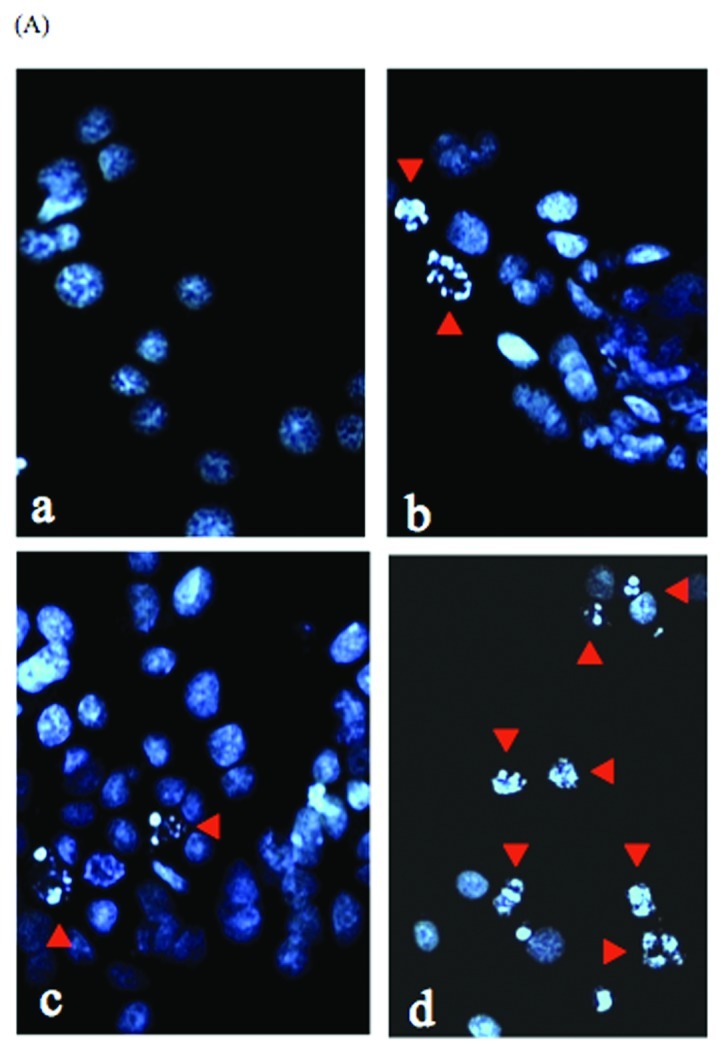

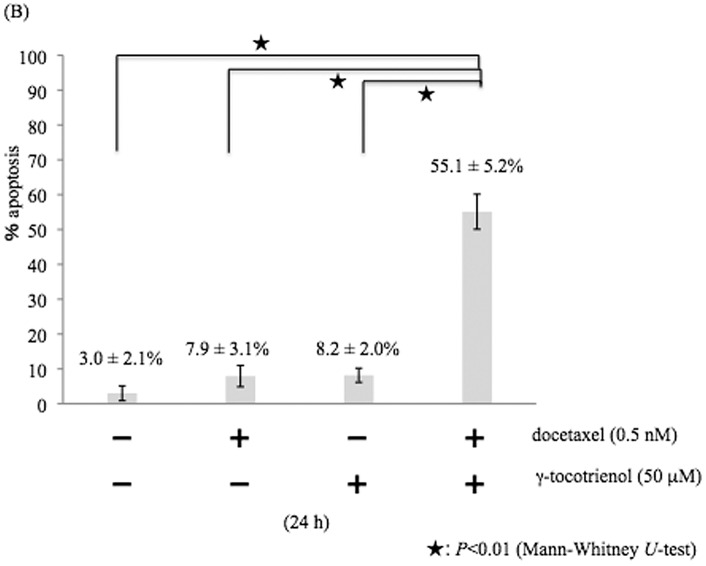
(A) *In vitro* morphogenetic behavior of cells (a) untreated or those treated with either (b) docetaxel (0.5 nM), (c) γ-tocotrienol (50 μM), or (d) both agents for 24 h. (a-d) A photomicrograph of Hoechst 33258-stained B88 nuclei revealed the chromatin condensation and nuclear fragmentation (red arrow heads) typical of apoptosis. (Original magnification, ×100.) (B) Effect of docetaxel (0.5 nM) and γ-tocotrienol (50 μM) on apoptotic cell death. Data represent the mean apoptotic cell death ± SD as follows: untreated control cells, 3.0±2.1%; docetaxel (0.5 nM)-treated cells, 7.9±3.1%; γ-tocotrienol (50 μM)-treated cells, 8.2±2.0%; and docetaxel (0.5 nM)- and γ-tocotrienol (50 μM)-treated cells, 55.1±5.2%. Results were analyzed by the Mann-Whitney U test. P<0.01 compared with untreated cells or cells treated with either docetaxel or γ-tocotrienol alone.

A photomicrograph of Hoechst 33258-stained B88 nuclei, from floating cells obtained by treatment with docetaxel, γ-tocotrienol, or both for 24 h, exhibited chromatin condensation and nuclear fragmentation typical of apoptosis (Fig. 7A). The percentages of apoptotic cells were 3.0% in the untreated control cells (Fig. 7B), 7.9% in the docetaxel-treated cells (Fig. 7B), 8.2% in the γ-tocotrienol-treated cells (Fig. 7B), and 55.1% in the cells treated with both agents (Fig. 7B).

## Discussion

Although chemotherapy and radiation therapy have proven to be highly effective in eradicating early oral SCC, the successful management of advanced (stage III and IV) oral SCC remains disappointing in the majority of cases (31). The poor clinical response in patients with advanced oral SCC has prompted the investigation of molecular targets for chemotherapy and radiotherapy in the treatment of oral cancer. Based on these considerations, we have previously analyzed the possible molecules responsible for the proliferation, invasion and metastasis of cancer cells, and have shown that human head and neck cells in oral squamous and salivary gland carcinomas have a significantly augmented NF-κB activity as compared to their normal counterparts (23,32), suggesting that NF-κB may be an important therapeutic target for the improvement of conventional chemotherapy in oral SCC. Thus, novel agents that are non-toxic, and can significantly inhibit constitutive and inducible NF-κB activation, as well as NF-κB-regulated gene products could remarkably augment chemotherapeutic drug-induced apoptosis. Therefore, the objective of the present study was to investigate whether or not γ-tocotrienol, a component of vitamin E, can enhance the antitumor activity of docetaxel against human oral cancer cells. The results showed that γ-tocotrienol suppressed the proliferation of human oral cancer cells, potentiated docetaxel-induced apoptosis, and inhibited constitutively active and inducible NF-κB activation, as well as the expression of NF-κB-regulated gene products. Our results are in partial agreement with those from other studies (33,34), reporting that γ-tocotrienol suppresses the proliferation of gastric and pancreatic cancer cells.

Specifically, we found for the first time that a low dose of γ-tocotrienol, when used in combination with docetaxel at a non-cytotoxic dose, is highly effective in inducing apoptosis in oral cancer cells. This is a very important finding as, although γ-tocotrienol has previously been suggested to induce apoptosis in gastric and pancreatic cancer cells (33,34.), its effect in combination with chemotherapeutic agents, such as docetaxel has not been previously investigated in human oral cancer. In this study, we demonstrate that this effect may be exerted through the downregulation of NF-κB-mediated survival proteins, such as survivin, Bcl-2, cIAP-1, cIAP-2 and XIAP by γ-tocotrienol. Moreover, since we have previously shown that constitutive and inducible NF-κB expressed in human oral cancer cells has been linked with chemoresistance (17), the suppression of NF-κB activity by γ-tocotrienol may account for the enhanced chemosensitivity of oral cancer cells to docetaxel.

In the present study, we report that even a non-cytotoxic dose of docetaxel can induce NF-κB DNA-binding activity in oral cancer cells, thus raising the possibility that cytotoxic doses of docetaxel used in the clinical setting could stimulate, to a greater degree, NF-κB activity in cancer cells. This possibility may significantly limit the antitumor activity of docetaxel in patients with oral cancer. On the other hand, γ-tocotrienol decreased NF-κB-binding activity in B88 cells. It is noteworthy that γ-tocotrienol alone had minimal effects on cell viability, whereas the combination with docetaxel increased antitumor activity with respect to chemotherapy alone in oral cancer cells. Although the effects of γ-tocotrienol in tumor cells are not yet fully understood, its abilities to induce cell cycle arrest (35), activate p53 (36), activate caspase-8 (21), suppress adhesion molecules (37), downregulate c-Myc and telomerase (38) and inhibit angiogenesis (39) have been suggested. Since the NF-κB pathway plays a critical role in tumorigenesis, chemosensitization, apoptosis, cell adhesion, the expression of c-Myc and human telomerase reverse transcriptase and cell cycle arrest (40), γ-tocotrienol must therefore modulate these pathways and molecules by suppressing NF-κB activity.

Based on the fact that the activation of caspase-3, an executioner caspase in the apoptotic pathway, leads to the cleavage of PARP in B88 cells (16), analysis of the conversion of procaspase-3 to caspase-3 by docetaxel and γ-tocotrienol is indeed important in any evaluation of apoptotic cell death. Consistent with this premise, the degree of growth suppression by these agents correlated well with the activation status of caspase-3 in B88 cells, indicating that caspase-3 targets cellular proteins, such as PARP and DNA fragmentation factor (41), for proteolytic cleavage resulting in cell death (42).

In conclusion, the results of the present study indicate that γ-tocotrienol potentiates the anticancer activity of docetaxel by downregulating the expression of NF-κB and NF-κB-regulated gene products, leading to the inhibition of proliferation. A number of clinical trials with tocotrienols in patients with pancreatic, prostate and breast cancer are already in progress. Based on our present results, well-designed animal and clinical studies are required for the potential translation of our preclinical findings in patients with oral cancer.
